# Relatives’ perspectives on encounters and communication in nursing homes during the Covid-19 pandemic: a qualitative interview study

**DOI:** 10.1186/s12877-022-03364-1

**Published:** 2022-08-25

**Authors:** Elisabet Eriksson, Katarina Hjelm

**Affiliations:** 1 Department of Public Health and Caring Sciences, Uppsala University, Box 564, 751 22 Uppsala, Sweden; 2 Faculty of Health and Occupational Studies, University of Gävle, 801 76 Gävle, Sweden

**Keywords:** Qualitative study, Covid-19, Family members, Cross-cultural communication, Nursing homes

## Abstract

**Background:**

Relatives of nursing home (NH) residents have experienced national or local restrictions on visiting their elderly family member during the Covid-19 pandemic. Today, many NHs have a multicultural environment, as staff, residents and their relatives speak different languages. Thus far, studies of remote communication with staff with limited language skills during the Covid-19 pandemic are lacking.

**Aim:**

The aim of the present study was to explore relatives’ experiences of encounters and communication with staff and residents in NHs during the Covid-19 pandemic.

**Method:**

An explorative qualitative study using semi-structured telephone interviews with 17 relatives of NH residents (12 women and 5 men). Data were analyzed using qualitative content analysis to identify four main categories and nine sub-categories.

**Results:**

Communicating during visiting restrictions was challenging, and relatives experienced ups and downs when trying to stay in contact with NH residents and staff. Relatives received general information, but desired information about residents’ everyday life without having to ask for it. Moreover, remote communication was difficult for residents with hearing impairment or dementia. Even relatives who understood different languages had to develop strategies to communicate with staff with limited language skills in Swedish and English. Relatives did not mention using translating applications to facilitate communication.

**Conclusion:**

During visiting restrictions, relatives lacked information about residents’ everyday life and found communication with staff with limited language skills challenging. For this reason, and to enable communication with staff with limited language skills, there is a need to find practical and technical solutions for facilitating remote communication between relatives, residents and staff at NHs.

**Supplementary Information:**

The online version contains supplementary material available at 10.1186/s12877-022-03364-1.

## Background

Relatives' everyday lives were suddenly affected when visiting nursing homes (NHs) was not possible due to national restrictions during the Covid-19 pandemic in the spring of 2020 in Sweden [1]. Many NHs today have a multicultural environment, as staff, residents and their relatives have different native languages and cultural backgrounds due to increased global migration [2]. Sweden, like many other European countries, has thus become multicultural; about 20 percent of the population was born abroad and more than 200 different nationalities are represented [3]. Like in many countries, the increased demand for workers has contributed to increasing the proportion of foreign-born staff in elderly care in Sweden [4]. In 2019, almost one third of assistant nurses working in home care and NHs were born abroad, most of them coming from Asia, Africa and Europe (not including the Nordic countries) [5]. Although studies of remote communication during the Covid-19 pandemic exist [6], studies on how communication in a multicultural society has been affected by the pandemic are lacking.

Close family members, husbands, wives, spouse, partners, sons, daughters, and grandchildren (hereafter referred to as relatives) are important to NH residents’ wellbeing. Relatives are involved in several ways in NHs. They assume different roles when visiting the elderly, e.g., providing hands-on assistance [7], overseeing the quality of care and providing supplies [7, 8], giving socioemotional support by replicating routines of importance to the elderly person, thereby teaching staff about the elderly resident’s preferences and life story, which helps staff understand the resident’s life context [7, 9].

There is evidence to suggest that relatives want to be informed and listened to, as this helps build trustful relationships between staff and relatives [7, 9]. Relatives expect clear, complete, and timely information on the resident’s condition, given by someone who knows the resident well [10]. Due to the visiting restrictions, relatives’ regular patterns of visiting NH residents abruptly ended, and relatives had to communicate in other ways with staff and residents. However, lack of physical visits should not hinder fruitful communication and engagement between relatives and residents [11]. Studies exploring communication during the Covid-19 pandemic have shown that NH visitors experienced loneliness and low psychological and emotional wellbeing [12], even though they could keep in contact with residents. Greater phone frequency was associated with a lower level of negative emotions for family members and friends [13]. In Sweden, few relatives of an elderly person who passed away in a NH during the pandemic were able to be present at the time of death [14]. Studies investigating remote communication during this time have reported that family members rated the overall experience of end-of-life care better when effective remote communication was used with both the patient and staff [15]. Similar findings, showing that staff availability for remote communication and being kept informed about the patient’s condition and care plan were important parts of high-quality communication [6]. Moreover, a Dutch study report that relatives of NH residents were most satisfied when they could stay in contact with the elderly person via telephone, behind glass, or outside at a distance [16]. Staying connected with residents is important for relatives, even though enabling this communication may be challenging for NH staff [17].

Stay connected during the Covid-19 pandemic may be even more challenging when residents, relatives and staff have different native languages. Immigrant nursing staff report limited language skills as one mayor problem in their work in the new host country, as it affects their ability to interact with patients, relatives and their colleagues [18, 19]. Knowledge concerning how communication works within multicultural elderly care settings exists, but we do not know how this communication has been affected by the consequences of the pandemic, e.g., staff and relatives wearing masks, visors, and maintaining a distance from residents. Previous studies have shown that relatives of residents from other countries provide psychological support to help residents create a sense of home and maintain their self-worth in NHs [8]. Relatives can also provide support by helping to develop individual communication resources (booklets) that improve staff’s communication with the elderly person and by being available if communication is not working [20, 21]. However, research on how relatives interact with staff with limited language skills is lacking.

People find ways to stay in contact with loved ones even in unpredictable life situations such as the Covid-19 pandemic. Thus, we need to know more about what methods relatives find fruitful for communicating with NH residents and care staff when physical visits are not possible. The present study aims to explore relatives’ experiences of encounters and communication with staff and residents, mainly through remote communication during the restrictions. We need to understand relatives' perspectives on encounters and communication methods during the Covid-19 pandemic if we are to understand, learn about and prepare for similar circumstances in the future.

## Method

### Design

An explorative qualitative study design was used. Semi-structured interviews were selected for data collection to obtain in-depth knowledge about the studied phenomenon, as such interviews provide a framework for the conversation and enable follow-up questions that probe deeper into respondents’ thoughts [22].

### Setting

The study was conducted in a county in central Sweden with eight municipalities and approximately 380,000 inhabitants. In the county, NHs are run mainly by the municipalities, but also by private companies. These NHs can have one to several wards for elderly people, and some wards are devoted to elderly people with dementia.

During the Covid-19 pandemic, national restrictions on visiting elderly people in NHs were introduced by the Public Health Agency of Sweden from April 1, 2020, to September 30, 2020. Thereafter, municipalities have been able to place temporary restrictions on NH visits [1]. When restrictions ended, NH management were strongly encouraged to set up routines for visiting elderly persons to minimize congestion and transmission of infection. In addition to the restrictions, during the pandemic in 2020 and 2021, various other measures were taken to prevent the spread of infection, e.g., using plexiglass between visitors and residents, masks, visors and recommendations to keep a distance (1.5–2 m) from others. The group of elderly people living in NHs was, and still is, a priority group for vaccination against Covid-19. In Sweden, this group received Covid-19 vaccines from January to March 2021.

However, well-known structural deficiencies within Swedish elderly care – such as a fragmentated organization, lack of staff, inadequate skills and poor working conditions [23] – can explain why care for the elderly was severely affected during the pandemic. In the studied county, it was challenging to train staff in both hygiene routines and competence concerning basic care hygiene. The training was further often limited to regular staff and provided digitally, implying that staff with limited knowledge of Swedish had difficulties accessing the training and understanding the content [24].

### Participants and procedure

Purposive sampling was used to contact relatives of elderly people living in NHs. Operations managers for elderly care institutions in the eight municipalities were contacted to obtain permission to conduct the study. After approval, the first author contacted the first-line managers at the NHs (*n* = 57) by email and/or phone and informed them about the study. Six did not wish to participate due to the strained situation at the NH. The first-line managers who accepted the invitation then sent information about the study and a request to participate by email to relatives of elderly residents at the NHs. Some first-line managers placed information about the study in residents’ rooms. Of the 51 NHs receiving information about the study, nine participated; four of them were privately owned and five public NHs in two municipalities. Those who were interested in participating contacted the first author (who was the principal investigator) by post, phone or email with contact information; the first author then contacted the respondents to schedule a time for the interview.

Participants’ characteristics are presented in Table 1. The majority of the relatives were born in Sweden. They had different education levels and worked, or had worked, in various occupations within the healthcare, education, or business sector.

**Table 1:** Characteristics of the study population

Variable	Relatives *N* = 17
Age (years)^a^	67 (39–84)
**Gender**	
Male	5
Female	12
**Country of birth**	
Sweden	14
Denmark	1
Iceland	1
Guiana	1
**Level of education**	
Primary school (< 9 years)	1
Upper secondary school (≥ 9 years)	2
Education at university level < 2 years	5
Education at university level > 2 years	9
**Employment**	
Unemployed	1
On retirement pension	10
Working	
Full time	2
Part time	4
**Family status**	
Married	9
Cohabiting	5
Divorced/Widow	1/1
Living alone	1
**Relationship to the elderly person at the nursing home**	
Wife	4
Husband	1
Daughter	5
Son	4
Grandchild	1
Cohabitant	1
Close friend/legal guardian	1
**Number of languages reported to be understood**	
< 2	4
3–4	9
≥ 5	4
**Languages relatives reported they could understand**	
Swedish	17
English	17
German	10
French	5
Norwegian	5
Danish	5
Spanish	2
Italian	2
Icelandic	1
Greek	1
Latin	1
Sign language	1

^a^ Values are Median (Range)

### Data collection

Collection and analysis of data proceeded concurrently until no new information was added in the data analysis [22]. Semi-structured telephone interviews, held in Swedish, were conducted between November 2020 and June 2021 by a registered nurse (first author) experienced in both elderly care and interviewing migrant nurses and physicians working in Sweden [18]. The interviews were recorded on a MP3-player and lasted between 20–60 min (median about 30 min). An interview guide – developed by the researchers for the present study and based on literature and previous expertise in the field – was used and covered questions on: (1) participants’ sociodemographic background data, (2) communication with the elderly person at the NH, (3) communication with staff at the NH, including communication with staff with limited language skills in Swedish, (4) encounters and perceived attitudes of staff towards the elderly and relatives, (5), how the Covid-19 pandemic may have affected their communication/relationship with their elderly family member at the NH, and (6) communication with care staff (supplement file). Additional, probing questions was used for example, ‘Can you give an example of a situation when staff could not understand you? The authors had no relationship with the participants, operation managers or the management at the NHs prior to data collection. A pilot interview (included in the study) was conducted, and minor corrections were made to the interview guide.

### Data analysis

Qualitative content analysis was applied [22] and data handled inductively [25] The interviews were transcribed verbatim (by a professional secretary) and each interview was given an identification number. The first author began by reading through the transcripts to get a sense of the whole and each interview was considered a unit for the analysis. The transcripts were exported to Open Code [26], a computer application that assists with labelling and organizing data. Phrases and text that were related to the study aim were identified thus meaning units were marked in the transcripts by the first author. The meaning units were thereafter coded and entitled as close to the text as possible [22]. The codes were read several times to find similarities and differences between them, and groups of codes sharing common attributes were organized into sub-categories and categories and labelled by the first author.

Data collection and data analysis proceeded concurrently until no new data was added in the analysis. The first author took fieldnotes during the interviews, performed the data analysis and both authors coded and compared one transcript. The second author checked the content of the coded data in relation to categories and subcategories [22]. Both authors reflected on and discussed the code labels and category names, disagreements regarding interpretation and labelling of codes and categories were solved through discussion until consensus was reached. Credibility was further strengthened by use of purposive sampling, entailing that the sample varied in age, gender, profession and kind of relationship with the elderly NH resident [22]. Moreover, the data analysis was performed by two registered nurses and researchers in Nursing Science, one of whom had vast experience of the area of migration and health and qualitative research methods (second author). To enhance dependability, an interview guide was used to ensure that the participants were asked the same questions and only one interviewer conducted the interviews. To enable transferability, the participants’ characteristics and information on the setting are provided. Confirmability was achieved by exemplifying each presented category with quotes from the participants’ responses.

### Ethical considerations

The Swedish Ethical Review Authority approved the study (Reg. no. 2020–03636). All participants received written information about the study by email prior to the interview. Before the interview started, they received oral information and had time to ask questions; they gave their written and verbal informed consent to participate. Participation was voluntary, and participants were informed that they could withdraw from the study at any time, without any negative consequences for their relative at the NH or themselves. Transcripts were deidentified, coded and digitally stored in a space intended for research data.

## Results

The following main categories emerged from the data analysis: experiencing kind encounters with staff but worries about residents’ wellbeing, feeling disconnected and connected during visiting restrictions, experiencing hampered communication and limited information during visiting restrictions, and managing communication and cultural dissimilarities. These categories and their respective subcategories are presented in Table 2. In the following section, each category is described along with its subcategories and illuminating quotes from the interviews.

**Table 2:** Overview of categories and subcategories

Category	Sub-categories
Experiencing kind encounters with staff but worries about residents’ wellbeing	• Satisfaction with interactions with nursing home staff
	• Experiencing worries linked to staff and nurses' abilities, and knowledge
Feeling disconnected and connected during visiting restrictions	• Experiencing ups and downs during visiting restrictions
	• Trying to maintain contact to avoid loneliness among elderly
Experiencing hampered communication and limited information during visiting restrictions	• Experiencing lack of information and both efficient and inefficient communication with staff
	• Impaired communication even if you adapt yourself and communication channels to the elderly
Managing communication and cultural dissimilarities	• Experiencing restricted communication with staff with limited language proficiency
	• Facing and handling misunderstandings and cultural differences
	• Finding strategies to communicate with staff with limited language proficiency

### Experiencing kind encounters with staff but worries about residents’ wellbeing

The relatives reported having had mostly positive experiences from interacting with NH staff and management. Most of them had also noted and felt that nursing staff at the NH were kind to the elderly, while some were less satisfied with temporary staff.

#### Satisfaction with interactions with nursing home staff

The relatives reported being generally satisfied when interacting with NH managers and care staff. They talked about the staff as being nice, calm, kind, and even very kind and wonderful. Another relative turned it around, stating that there had been no discussions between relatives about staff being unkind. Further, staff were helpful and regular staff were happy and positive when they visited the NH. A few reported that staff were fairly good or that not all staff were good. However, relatives mainly reported having a good relationship and getting along with the NH staff.

I just think that when there is something and I call and ask if we can come by or whatever. It’s always fun to call, I think. I know I’ll be treated well (R 9)

#### Experiencing worries linked to staff and nurses' abilities, and knowledge

Generally, relatives reported that staff were kind to the elderly and took good care of them. However, participants made a distinction between regular and temporary staff, where the former were more caring towards residents and joked with them. According to the participants, temporary staff had less knowledge and information than regular staff, and some participants did not ask temporary staff questions about the resident’s health status and care. Due to the lack of knowledge and skills among temporary staff, a few relatives felt temporary staff should be offered courses to enable them to provide higher-quality elderly care.

Some mentioned that staff, whether regular or temporary, did not have time to sit down and talk with NH residents and described the elderly persons as being very lonely. Some relatives also complained about staff behavior. For example, staff could forget things, e.g., to get the elderly person out of bed, leaving them alone without an alarm, and not taking them outdoors. Such incidents created worries about the elderly person’s wellbeing when relatives could not visit the NH. Some relatives reported being very angry when they witnessed staff sitting down for long talks and breaks, while the elderly were not taken to outdoor activities, like music events in the garden.

And she says “they’re so nice here” [laughter]. “They take such good care of me.” (R 4)

And it also happens that they (residents) sit alone on the balcony… leaving them alone on the balcony is pretty sloppy. Not that I think they’d kill themselves, but they could fall. (R 16)

” I was going to pick him up, and he’d been lying in bed awake for a pretty long time. He usually sleeps two hours in the afternoon. And he tried to get out of bed himself. They’d forgotten to get him ready. (R 14)

### Feeling disconnected and connected during visiting restrictions

The relatives reported having experienced ups and downs during the visiting restriction and trying to maintain contact with their elderly family member. Most of them had felt disconnected from the resident during this time, but also happiness once they could see the elderly person again.

#### Experiencing ups and downs during visiting restrictions

Visiting restrictions came abruptly for the relatives, and it was tough when restrictions suddenly changed. This was described as stressful and frustrating; the world had turned upside down and some felt sad and powerless. Visiting restrictions also contributed to anxiety about suffering and concern about not being present if the resident were to die.

All mentioned that the frequency of visits was lower, and a few had not seen their elderly family member for more than a year. Relatives had been hesitant about visiting the NH due to fear of increased risk for infection for the residents or themselves, but also because of mixed messages about visiting restrictions from the NH staff. Booking visits was tiring, and relatives often had to contact the first-line managers to get approval for a visit. For many participants, seeing each other was valuable and joyful; they had taken walks and enjoyed drinking coffee together. Others reported that the lack of physical contact felt strange and worthless. For example, sitting behind plexiglass or at two meters distance was heartbreaking, as was trying to keep the elderly person away from them when he/she wanted to come close. It was especially difficult for participants to explain to a family member with dementia why they could not come close. One consequence of the restrictions was that staff had to take over practical chores the relatives usually did at the NH, such as changing curtains in the resident’s room, watering flowers, foot care and cutting fingernails. Some relatives felt lonely when they could not continue their visits to the NH, while others thought it was a relief.

Well, plexiglass, sure, but… in our case anyway it was a pretty worthless form of communication. It was more like we could just see that she was there. (R 11)

#### Trying to maintain contact to avoid loneliness among elderly

According to the relatives, the visiting restrictions had a wide range of consequences for the residents; they were lonelier, sadder, had fewer activities at the NH and they were restricted in where they could go. Some relatives had worries about the elderly being “locked” in their rooms during outbreaks of Covid-19 infection at the NH and how that might affect them. Some reported having maintained the relationship with the resident through phone calls and visits, but for others the relationship was affected negatively. Relatives said they had tried to maintain contact without visiting the NH, and some became anxious once they were allowed to visit their elderly family member again.

So Mom was completely isolated and visits were restricted so we could only communicate over the telephone, and it’s very traumatic to move from your house to a nursing home and not be able to do that with your closest relatives nearby, who could visit. That makes it extra difficult. (R 3)

### Experiencing hampered communication and limited information during visiting restrictions

Relatives described the general information from the NH as mostly good, but they lacked information on the elderly person’s wellbeing and everyday life. They had changed communication methods, such as increased use of phone calls, but this did not work when communicating with elderly persons with hearing loss and dementia.

#### Experiencing lack of information and both efficient and inefficient communication with staff

Relatives had various experiences of getting information from the NHs and communicating with NH staff during the visiting restrictions. Many were satisfied with the information they received through weekly emails from the managers, while others received information monthly or more seldom. According to the relatives, the emails were informative, up-to date, and included practical information. A few felt the information was only general information from the municipality that the manager had made a few changes to. These emails were less appreciated by the relatives. However, almost all felt confident that they would get information from the NH if something very serious were to happen to their elderly family member. However, many relatives reported wanting more information and more contact with nursing staff, without having to ask for it. Some felt they chased staff on the phone or by email; they missed the small talk with staff and the everyday information concerning the elderly person. Hindrances to communication were several: staff did not have individual phones, the wrong staff answered, new staff did not have information, the relatives did not want to disturb the staff, or the staff did not have time due to their demanding workload. A few relatives had received calls from the physician regarding treatment when their elderly family member contracted Covid-19, and they felt the information was good. However, one relative felt pressed to approve palliative care (which meant not sending the elderly person to the hospital), which was very upsetting.

“I guess I think that, overall, communication from NN (name of the NH) has been pretty bad. I think that… Under the circumstance getting a happy information letter from the manager once a month isn’t enough”. (R 10)

#### Impaired communication even if you adapt yourself and communication channels to the elderly

All had communicated by phone during this period, and some were dependent on staff assisting the resident with using the phone. Several relatives were satisfied with communicating by phone, others described having no physical contact as useless, especially for residents with hearing loss or dementia. Only a few had used Skype/Facetime successfully; for others it was not possible because residents and staff had limited knowledge about how to use these applications. A few had written letters or sent email to residents. During the summertime, almost all had visited the NH to talk to their elderly family member outdoors or through a window. Because of the distance, some could not talk to the elderly person, and they found themselves having monologues, singing songs, or engaging in one-way communication. Regarding protective equipment, participants had used masks and sometimes both a mask and a visor, but found that this hampered communication with residents, who could not hear them or read their lips.

(call) No so often, talking on the telephone is difficult. I’m trying to get ahold of better… well, it’s hard to talk on the telephone, get her hearing aid adjusted, you know. She has a hard time managing, it works sometimes, but not others. But talking with her when you’re there, that works really well. (R 4)

And then they called so you could see [name] too, so they said they would… we would do it that way. But it’s hard for them to understand when they just see an image. And I saw [name], so surprise and he looked around and he heard me talking, he saw me on the screen and… But still, it’s hard for this particular category to understand, I think, all this modern technology. (R17)

### Managing communication and cultural dissimilarities

According to the relatives, communication with staff with limited language skills was challenging, as these staff members lacked nuances when they spoke Swedish. Furthermore, the participants reported experiencing cultural differences and having to find strategies to communicate with staff with limited language skills.

#### Experiencing restricted communication with staff with limited language proficiency

The relatives reported various experiences when communicating with staff with limited Swedish language skills. Talking to staff with limited language skills worked out well most of the time, especially in physical encounters, where body language facilitated communication. However, talking on the phone with them was described as much more difficult. Although, staff with limited language skills were nice to the participants and residents, several problems were mentioned. Staff and relatives had difficulties understanding each other; some staff members did not understand what the relatives said, staff spoke unclearly, expressed themselves inappropriately, written communication was a challenge, and communication was limited and awkward. No one mentioned using translating applications or having a translator present when speaking to staff with limited language skills. Relatives reported missing the nuances in the communication, stating that Swedish staff could tell them more about the residents. Relatives said they wished the staff spoke more fluently in Swedish and that communication was easier. They further noted that it is easier for them to talk to staff with limited language skills than to some residents who have impaired hearing. They thought that limited language skills among staff was confusing for the residents and that residents need to have a great deal of patience.

I got an answer when I asked how he was feeling. They said “yeah, but he is fine and he is alert.” The other girl (Swedish staff) answered… she could say more: “yes, but he got up eventually. He was a little tired today and wanted to sleep a bit longer. So he got to stay in bed.” I don’t think I would have gotten that information from the other girls. (R 1)

#### Facing and handling misunderstandings and cultural differences

In the interviews, few mentioned the occurrence of language-based misunderstandings with NH staff. However, answering a direct question, several relatives described mix-ups with staff with limited language skills. Although misunderstandings were not uncommon, things seemed to work out most of the time. Misunderstandings were described as minor, or no big deal, even when the relatives did not get what they wanted, e.g., were not served the drink they had requested. Cultural differences were also mentioned in relation to staff with limited language skills. One relative described how a joke had made staff very angry. This occurred when he/she took down a Christmas advertisement in March and said it was high time; the incident had taken time to sort out. Other informants had a variety of experiences of foreign staff. These staff members were described as being very needed, being nice, having respect for the elderly, and being talented, but it was also reported that residents could be afraid of them. A few said that these staff were not as good as native staff or even that they were mean to residents.

The language issue is difficult. Even those who have lived in Sweden a long time can really misunderstands things a lot. (R 16)

I mean, it’s not just language, it’s also a bit… Anyway, somehow there’s a clash of cultures. (R 1)

##### Finding strategies to communicate with staff with limited language proficiency

The relatives talked about the strategies used when they had difficulties communicating with staff with limited language skill; these included continuing to speak Swedish instead of using English, as they were afraid using English would confuse staff even more, but some used some English words when talking. Relatives described how they spoke clearly, used simple language, replaced words, put the verb last in the sentence, changed the word order, adjusted the communication to the staff’s linguistic level and choose/selected what to talk about. When they adjusted to staff members’ linguistic level, some relatives felt they could neither explain what they meant nor be themselves. Quite often, staff with limited language skills asked a colleague to call the relative back. Some relatives wrote to staff instead of calling them or tried to contact other staff members, for example the nurse, the resident’s contact person, or the manager. Some used gestures, and relatives sometimes chose not to talk to staff with limited langue skills or called back later, hoping someone else would answer the phone. A few had observed residents turning to other Swedish-speaking staff.

Well, I hear who’s answered when I call, and then I can choose what I bring up and I can email the nurse instead, or I can email Mom’s contact person too. (R 13)

… but where there are temporary staff who have a hard time understanding over the phone, then…But they usually give the phone to someone who can talk (R 9)

Well you know, I don’t think they could speak English either, to be honest. It’s stayed at that level. Sometimes I haven’t been able to explain what I mean. The level has been that low. (R 8)

## Discussion

The present findings contribute in-depth knowledge about relatives’ experiences of communicating with NH residents and staff in a multicultural context during the Covid-19 pandemic. The relatives were generally satisfied when interacting with the NH staff, but were worried about the residents’ health and wellbeing when they could not visit them. Moreover, they experienced ups and downs when trying to maintain contact with residents in various ways and tried to prevent their elderly family members from experiencing loneliness. Although relatives felt certain they would get information from the NH in crucial situations, they lacked information about the resident’s everyday life. Furthermore, some struggled to keep connected to residents with hearing impairment or dementia, as speaking on the phone or using video meetings was very difficult for these groups of residents. Moreover, there was the challenge of communicating with staff with limited language skills, and relatives had developed and used various strategies to stay informed about the residents.

Relatives were satisfied when they received information from the NH, e.g., from managers and care staff, and when they could communicate with residents even though they could not visit the NH. These findings confirm previous research during the pandemic showing that, in severe situations, available staff and effective remote communication can compensate for physical visits [6, 16]. However, our findings revealed that relatives had to ask for information from the NHs, especially concerning the residents’ daily life, and that they preferred receiving this kind of information on regular basis, without having to ask for it. This indicates that structures for communication between relatives and staff can be improved. Some relatives had positive experiences of communication with a so-called contact person, while others were less satisfied with their contact person or did not have one. Thus, NH managers and leadership need to secure structured communication channels, especially during visiting restrictions, to enable residents and relatives to maintain contact and their relationship.

Our findings indicate that relatives tried to stay connected to residents, but communication was hampered for numerous reasons. Some reported that residents could not handle a phone or video meeting equipment, and sometimes their hearing aids were broken. In these situations, relatives were dependent on staff being able to assist the resident, but communication failed when staff were not available or lacked the skills needed to assist with technical equipment such as hearing aid and video meeting devices. The lack of staff in elderly care during the pandemic has been reported previously [23], and the increased number of temporary staff at this time may explain their lack of competence. However, technical skills could be improved among both regular and temporary staff, thus enabling them to assist with communication between relatives and residents. Furthermore, technical solutions for communication could be simplified to make it easier for elderly with disabilities to use them. If such solutions already exist, NHs need to know about and have access to them.

Relatives reported that, beyond the challenges of communicating during visiting restrictions, it was also difficult to communicate with staff with limited language skills in Swedish and English. None of the relatives mentioned using an interpreter or technical aids, like existing translation apps, when talking to staff with limited language skills. This is surprising, as both relatives and staff should have access to translating apps on their smart phones. Future studies should investigate the availability of language aids and hindrances to using them.

Some relatives also reported very limited Swedish language skills among foreign staff, saying that they could not explain themselves. This is alarming, as communication between residents, staff and relatives is essential to building trustful relationships between them [7, 9, 10]. Good communication is a prerequisite for developing the core of trans-cultural care: the encounter between the person and staff in a caring relationship aimed at achieving health [27]. Although relatives could speak and understand many languages, these languages did not match staff members' languages, as their origins were in other cultures and countries. Furthermore, some of these staff could not speak English fluently, meaning there was no common language between relatives and staff. Relatives also suggested that staff needed to improve their language skills, and the findings even revealed the need for increased cross-cultural understanding. A range of interventions can help staff improve their language skills. An educational package for use in cross-cultural residential elderly care facilities has been developed [28] and a nurse-led cross-cultural care program has been reported to improve aged care workers' cultural competence [29]. The Covid-19 pandemic has shed light on the need for language proficiency among elderly care staff. Hopefully, increased use of cross-cultural and language education packages and interventions will be seen in the future.

This research adds the necessity to emphasize improvement of communication between NH staff and relatives, considering lessons learned during COVID-19 pandemic.

### Strengths and limitations

Telephone interviews were conducted instead of face-to-face interviews due to the pandemic, and sometimes it is difficult to communicate by phone compared to in person. This may also have influenced who participated, in that relatives who prefer face-to-face meetings and those with hearing impairments may have chosen not to participate. The majority of participants were born in Sweden, although our intention was to include relatives born in different countries. Information about the study was emailed through the first-line manager at the NH to relatives, and those interested contacted the researcher. If the researcher had provided information about the study at the NH in person, more participants from other countries may have volunteered. The strength of the present study is that it deepens our understanding [22] of relatives’ experiences of communication during visiting restrictions. A thorough description of the audit trail, including data collection, the analyses and quotes from participants help the reader transfer the results to settings or participants with similar characteristics [22].

## Conclusion

The findings show that upholding relationships with loved ones living in NHs has been challenging during visiting restrictions, e.g., during a pandemic such as Covid-19, and that relatives experienced ups and downs when trying to maintain contact with residents. Relatives generally felt certain they would receive information from the NH in critical situations, however they wanted to receive more information about residents’ everyday life, without needing to ask for it. Moreover, even though relatives were fluent in many languages, these languages were not the same as those spoken by staff. Relatives called for improved Swedish language skills among caring staff. This indicates that, to enable communication with staff with limited language skills, there is a need to find practical and technical solutions for facilitating remote communication between relatives, residents and staff at NHs.

## Supplementary Information

**Additional file 1. **Topic guide.

## Data Availability

The datasets are not available from the corresponding author on request due to reasons concerning the protection of participants’ privacy and confidentiality.
