# Supplementary material for: Relatives’ perspectives on encounters and communication in nursing homes during the Covid-19 pandemic: a qualitative interview study
Source: BMC Geriatr. 2022 Aug 25;22:706. doi: 10.1186/s12877-022-03364-1 (PMC9409621; doi:10.1186/s12877-022-03364-1)
Supplement: Supplementary file 1 — Additional file 1. Topic guide. [file 12877_2022_3364_MOESM1_ESM.docx]

**Topic guide**

*Background data:*

How old are you?

Would you describe yourself as a Man □ Woman □ otherwise?

What is your country of birth?

How many years have you attended school?

□ Primary school (<9 years)

□ Upper secondary school ( ≥ 9 years)

□ Education at university level <2 years

□ Education at university level >2 years

□ Other education?

Are you employed?

□ Unemployed

□ On retirement pension

□ Working

□ Full time

□ Part time

What are you working with?

Are you:

□ Married

□ Cohabiting

□ Divorced/Widow

□ Living alone

□ Other

Which languages can you understand?

What is your relationship to the elderly person at the nursing home?

How do you think your relative are doing at the nursing home?

Is there anything that is especially good / bad at the nursing home?

*Communication with the elderly person at the NH*

How do you usually communicate with your relative at the nursing home?

*Communication with staff at the NH, including communication with staff with limited language skills in Swedish*

How is it to talk to the staff?

Is it easy to talk / communicate with the staff? If not, can you tell me why?

Does the staff speak a language other than Swedish with you? Do you know which ones?

How do you communicate with the staff if it is difficult to understand what they are saying?

Can misunderstandings arise if it is difficult to understand the staff, can you give an example?

How do you handle the situation when there is a misunderstanding?

*Encounters and perceived attitudes of staff towards the elderly and relatives,*

How would you describe the relationship between you and the healthcare staff?

How would you describe the relationship between your relative and the healthcare staff?

Is there something you wish was different or that you would like to change? If yes, what?

*Has the Covid-19 pandemic affected communication/relationship with elderly family member at the NH*

Can you tell me how you got information about corona (covid-19)?

Can you tell me about how the corona pandemic may have affected communication to your relative at the nursing home?

Has the pandemic/visiting restrictions affected your relationship with your relatives?

*Communication with care staff during the pandemic.*

Can you tell me about how the corona pandemic may have affected communication with staff?

Do you think your relative get the care he/she need, or do you think something is lacking?

If yes, what?
